# Anorectal autotransplantation in a canine model: the first successful report in the short term with the non-laparotomy approach

**DOI:** 10.1038/srep06312

**Published:** 2014-09-10

**Authors:** Jun Araki, Yuji Nishizawa, Tatsuo Nakamura, Tomoyuki Sato, Munekazu Naito, Naoyuki Hatayama, Shuichi Hirai, Kensuke Tashiro, Isao Koshima

**Affiliations:** 1 Department of Plastic Surgery, University of Tokyo, Tokyo, Japan; 2 Department of Colorectal Surgery, National Cancer Center Hospital East, Chiba, Japan; 3 Department of Bioartificial Organs, Institute for Frontier Medical Science, Kyoto University, Kyoto, Japan; 4 Saitama Shinkaibashi Clinic, Saitama, Japan; 5 Department of Anatomy, Tokyo Medical University; 6 These authors contributed equally to this work.

## Abstract

Colostomy is conventional treatment for anal dysfunction. Recently, a few trials of anorectal transplantation in animals have been published as a potential alternative to colostomies; however, further development of this technique is required. In this study, we utilized a canine model of anorectal transplantation, evaluated the patency of our microsurgical anastomoses, and assessed the perfusion of the transplanted anus. We designed a canine anorectal transplantation model, wherein anorectal autotransplantation was performed in four healthy beagle dogs by anastomoses of the lower rectum, the bilateral pudendal arteries (PAs) and veins (PVs), and pudendal nerves (PNs). Postoperative graft perfusion was measured by indocyanine green (ICG) angiography and histological examination. The length of the anorectal graft including perianal skin, anal sphincter muscle, bilateral PAs, PVs, and PNs was 4.9 ± 0.3 cm. All diameters of the PAs, PVs, and PNs were large enough to be microscopically anastomosed. Both ICG angiography and histological examination demonstrated good graft perfusion, except for one case that lead to venous congestion. These results show that anastomosis of the bilateral PAs, PVs, and PNs is required for anorectal transplantation. This is the first successful report of canine anorectal autotransplantation.

Defecation is a major activity of daily living. It may be impaired because of congenital anal dysfunction caused by anal atresia, or Hirschsprung's disease, intractable anal fistula, and rectal or perianal cancer resections. In most of these cases, a colostomy is the primary treatment. Following publication of the pioneering report on abdominoperineal resection of the rectum by the British surgeon Miles in 1908, colostomies gained international acclaim throughout the 20th century^1^,^2^. The potential number of patients who currently undergo colostomies amounts to approximately 200,000 in Japan, 650,000 in Europe, and 700,000 in the U.S.^3^. Although this procedure is essential to prolong patient survival, it is accompanied by many problems such as troublesome postoperative management, cosmetic concerns, and psychological distress. Some patients prefer death to living with a stoma^4^, and psychosocial sequelae are inevitable in patients who have undergone ostomies as well as in patients who have lost their limb or breast^2^,^5^. To address these issues, some reconstructive methods including gracilis^6^ or gluteus maximus muscle transfer^7^, antropylorus transposition^8^, and artificial sphincter implantation^9^ have been reported to restore anal function. However, none of these are a ‘gold standard’ technique due to the complex nature of anal functions^10^. Meanwhile, owing to improvements in microsurgical technique, instruments, and immunosuppressive therapy, organ transplantation has gained great popularity worldwide. Transplantations of not only vital organs are popular. For example, vascularized composite tissue allotransplantation (VCA) including the upper^11^ and lower extremities^12^, face^13^, uterus^14^, and larynx^15^ are performed to improve patients' quality of life (QOL). Anorectal transplantation (i.e. of all organs involved in defecation, including the perineal skin, anus, rectum, and the sphincter muscle) has strong potential as an innovative therapy for anal dysfunction. Anorectal transplantation models using rats^16^,^17^ and swine^18^,^19^ have been reported in experimental trials; however, these experimental systems have considerable drawbacks. Because rats and pigs do not have the capacity to inhibit defecation and habits similar to those of humans, they are not particularly useful as model subjects for human application. Dogs living with humans and having defecation control are the most suitable of all laboratory animals for anal function evaluation. However a surgical technique of canine anorectal transplantation remains to be accomplished^20^. Therefore, in this study, we demonstrated a canine model of anorectal transplantation, evaluated the patency of our microsurgical anastomoses, and assessed the perfusion of the transplanted anus.

## Methods

All animal studies were performed according to the protocol approved by the Animal Care Committee and in accordance with the Animal Care Policies of Kyoto University in Kyoto, Japan.

### Animals

Five healthy adult beagle dogs weighing 6.0–11 kg were included in this study. Four healthy beagle dogs underwent anorectal autotransplantation, while the remaining dog was used for the control study involving ICG fluorescence angiography.

All surgical and euthanasia procedures were performed in accordance with the “Guide for the Care and Use of Laboratory Animals” published by the National Institutes of Health. The experimental protocol was approved by the Animal Experimental Committee of Kyoto University, and all efforts were made to minimize animal suffering. Fasting was imposed on all dogs for 24 h prior to the operation, but dogs were given drinking water *ad libitum*.

### Preparation for surgery

All surgical procedures and physiological measurements were performed under general anesthesia. The animals were premedicated by intramuscular administration of 0.05 mg/kg atropine sulfate. They were then anesthetized with 15 mg/kg ketamine hydrochloride and 3 mg/kg xylazine hydrochloride and intubated endotracheally. Continuous monitoring was performed by electrocardiography and oxygen saturation by reflectance oximetry using a sensor clipped to the ear. The perianal region was shaved and the animals were positioned in the supine position. The abdominal region was disinfected with 70% ethanol and iodine tincture, and covered with sterilized drapes. Sevoflurane (0.5%–1.0%) and nitrous oxide gas were used for maintenance of anesthesia during the procedure under mechanical ventilation.

### Surgical procedure

**Figure 1:**
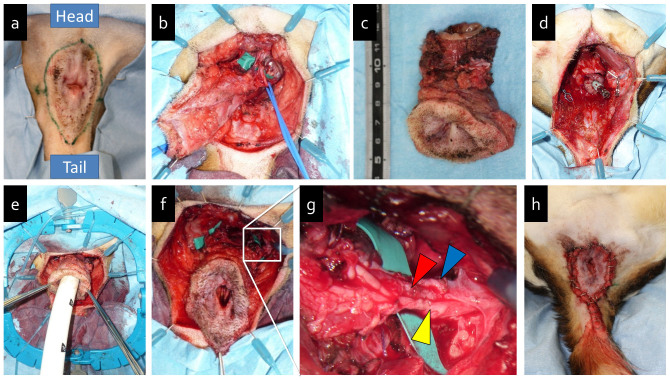
Canine anorectal autotransplantation procedure. (a). Preoperative design of circumanal incision. (b). Identification of pudendal artery (PA), vein (PV), and nerve (PN) (blue loop). (c). The harvested anorectal graft with preservation of the PAs, PVs, and PNs. (d). Proximal rectum stump before transplantation. (e). Rectal anastomosis with mechanical staplers. (f). Autologous transplanted anorectal graft. (g). Magnified view of microanastomoses of the left PA (red arrow), PV (blue arrow), and PN (yellow arrow). (h). Perineal closure.

Surgery was performed by a colorectal surgeon and a plastic surgeon. Following the circumanal incision (Fig. 1a), the anal canal was circumferentially dissected outside of the external anal sphincter muscle. Pudendal arteries (PAs), pudendal veins (PVs), and pudendal nerves (PNs) ran bilaterally along the inside of the ischial tuberosity and reached the external anal sphincter muscle at the 2 o'clock and 10 o'clock positions (Fig. 1b). The posterior wall of the anal segment was separated from the anterior surface of the coccygeal muscle. The levator ani muscle was transected at the lateral and posterior wall, and the anterior wall was detached behind the prostate. The PAs, PVs, and PNs were clipped and cut to avoid any damage. The rectum was separated with a mesentery at the lower part (Fig. 1c). Finally, an anorectal graft was harvested (Fig. 1d).

The anorectal graft was transferred to the surgical field from the back table following perfusion with 20 ml heparinized saline. The rectal stump was mechanically anastomosed utilizing DST Series TM EEA TM Staplers (Covidien, New Haven, CT) (Fig. 1e). Then, the anorectal graft was orthotopically repositioned (Fig. 1f). The bilateral PAs, PVs, and PNs were end-to-end anastomosed using interrupted sutures (10-0 nylon, Johnson & Johnson, Tokyo, Japan) under a surgical microscope (OME-9000, Olympus Medical Systems Corporation, Japan) (Fig. 1g). Anastomoses of two arteries (PAs) and two veins (PVs) were used for reperfusion of the anorectal graft. Then, the pelvic floor muscles including the levator ani were reconstructed using 4-0 vicryl sutures (Atom vet's medical, Kyoto, Japan) and closed using skin staples (3M™ Precise™ Vista Skin Staplers, Medema T/A Omega Medical Supplies Ltd., UK) (Fig. 1h). After surgery, dogs were fed liquid food and intramuscular administration of an antibiotic (cefazolin sodium, 50 mg/kg/day) was initiated.

### ICG fluorescence angiography

ICG fluorescence angiography was performed to evaluate postoperative blood supply as described previously^21^,^22^. Briefly, 1.0 mL of ICG (0.5% diagnogreen; Daiichi Pharmaceutical, Tokyo, Japan) was intravenously injected via a peripheral venous line. Intravenously injected ICG binds to circulating globulins and remains in the intravascular region. After binding to globulins, ICG absorbs light in the near-infrared range with a maximum of 805 nm and fluoresces with a maximum of 840 nm in plasma. We utilized a newly developed near-infrared camera system (PDE-neo™; Hamamatsu Photonics KK, Shizuoka, Japan) that activates ICG with emitted light (wavelength: 760 nm). An operator directly handled the camera unit of the device and observed real-time images on the monitor. These videos were analyzed using region of interest software (Hamamatsu Photonics K.K., Shizuoka, Japan). Brightness was divided into a 0–250 range using the software, and the mean brightness in red (positive control), blue (negative control), yellow (anal canal), and green (perianal skin) squares was measured over time. ICG fluorescence angiography was performed to evaluate blood supply of implanted grafts after surgery.

### Histological examination

Anorectal tissues were cut in the sagittal plane and fixed in 20% buffered formaldehyde for two days. After dehydration with ethanol, the tissues were embedded in paraffin. Then, 6 μm thick sections were cut in the coronal plane and hematoxylin and eosin staining was performed. In addition, 6 μm thick sections were cut in the horizontal plane and subjected to Masson's trichrome staining. Sections were cut at several levels and meticulously examined to identify any pathological findings. The rectum and anal canal areas, including mucous membranes and sphincter muscles, were examined. The degree of histological change was graded on a scale from 1–3 as follows: 1, no morphological change; 2, mild change and 3, severe change. Five sections of each sample were simultaneously evaluated as to the histopathological stage and graded by three senior histologists, who were unaware of the group each section was from. Results are described as means ± standard deviations.

## Results

### Time schedule and surgical parameters

The duration of each operative step and ischemic time were summarized in Table 1. Time taken for graft harvesting was 2 h 11 min ± 35 min (mean ± SD). A relatively long time was required to dissect the nerves and blood vessels carefully. The length of the anorectal graft including perianal skin, anal sphincter muscle, bilateral PAs, PVs, and PNs was 4.9 ± 0.3 cm. In the autotransplantation, the mechanical stapler was very useful because the proximal rectum stump was too short to be manually anastomosed (Fig. 1e). The diameters of the PAs, PVs, and PNs were measured under a surgical microscope. The diameters of the PAs, PVs, and PNs were 1.75 ± 0.3 mm, 2.5 ± 0.9 mm, and 1.75 ± 0.3 mm at the right, respectively, and 1.63 ± 0.3 mm, 2.1 ± 0.3 mm, and 2.4 ± 0.8 mm at the left, respectively (Table 1). All diameters were large enough to be microscopically anastomosed by plastic surgeons.

The duration of the anastomosis surgery was 3 h 23 min ± 34 min and the total ischemic period lasted for 4 h 37 min ± 34 min. Immediate blood flow through the graft occurred after anastomosis and removal of vascular clamps, with the graft transitioning from a white to red color concomitant with pulsations occurring through the pudendal artery and filling outflow veins. After confirming that blood flow had resumed in the transplanted anorectal graft, the pelvic floor muscles were reconstructed and closed. Total operation time was 8 h 5 min ± 45 min.

### Postoperative course in the short term

Animals were subcutaneously injected anti-inflammatory drugs twice a day for analgesia, observed in separate cages after surgery, and euthanized by overdose potassium chloride intravenous injection at experimental end point. The anorectal graft of dog 1 was macroscopically dark probably because of technical error. Therefore dog 1 was defined as an unsuccessful case, performed ICG fluorescence angiography and tissue biopsy, and sacrificed on the day following surgery. The other dogs observed for several days so far as the laboratory's circumstance and surgeon's schedule permits. Dog 2 and 3 were macroscopically confirmed their graft survival for 3 and 4 days after surgery and sacrificed. Dog 4 was observed for the longest 9 days, performed ICG fluorescence angiography and tissue biopsy, and sacrificed as a terminal euthanasia.

### ICG fluorescence angiography

**Figure 2:**
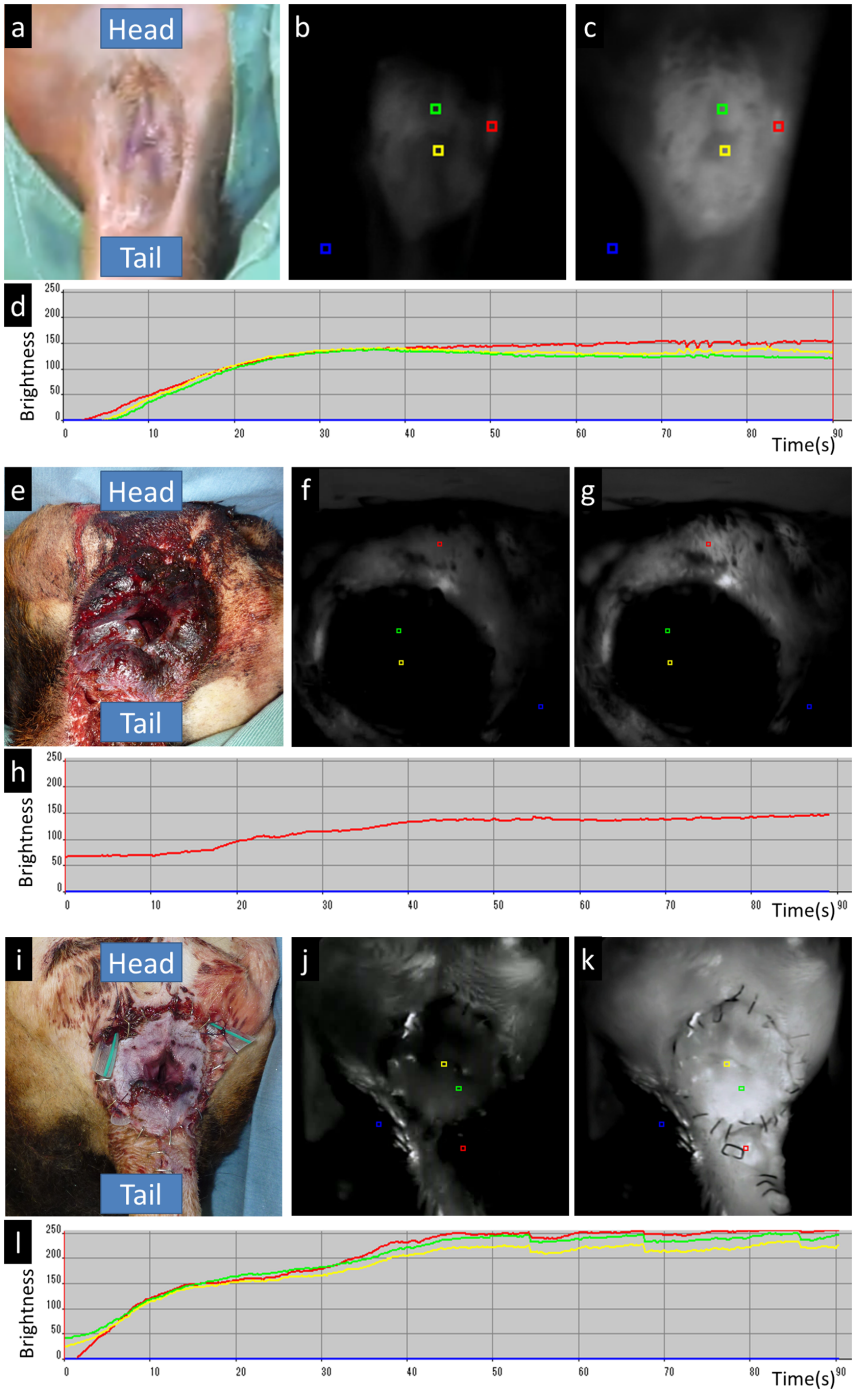
Postoperative indocyanine green fluorescence (ICG) angiography of the anorectal graft (normal dog: (a)–(d), dog 1: (e)–(h), and dog 4: (i)–(l)). (a), (e), and (i). Perineal view of the anal segment immediately before angiography. (b), (f), and (j). Beginning of enhancement of the anal segment. (c), (g), and (k). Plateau of enhancement. (d), (h), and (l). Time course of brightness determined using region of interest software (red line: positive control, blue line: negative control, yellow line: anal canal, and green line: perianal skin). X-axis is a time scale and Y-axis represents brightness of the tissue.

Blood flow of the transplanted graft was evaluated using ICG fluorescence angiography. The anorectal graft of dog 1 was macroscopically dark and examined on the day following surgery. The anorectal graft of dog 4 was examined nine days postoperation and defined as a successful case, and then both were compared with the normal dog. In the normal dog, the anal canal and perianal skin brightness rose in a similar pattern as that in the positive control and reached a plateau in approximately 30 s (Fig. 2a–d; also see Video S1). In dog 1, the anal canal and perianal skin brightness did not rise at all (Fig. 2e–h; also see Video S2). In dog 4, the anal canal and perianal skin brightness rose in a similar pattern as that in the positive control and reached a plateau in about 40 s (Fig. 2i–l; also see Video S3).

### Histological examination

**Figure 3:**
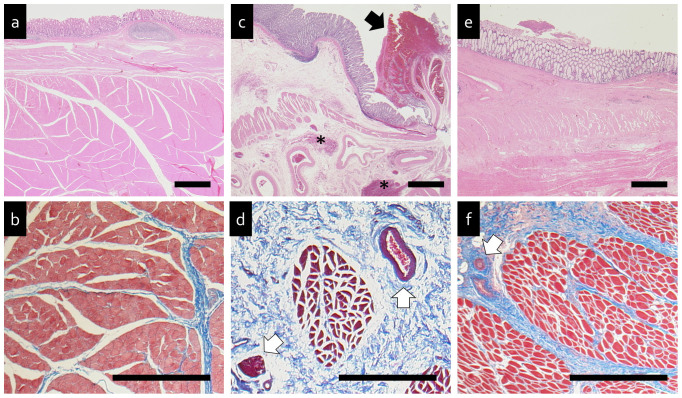
Histological examination (normal dog: (a), (b), dog 1: (c), (d), and dog 4: (e), (f)). (a), (c), and (e). Hematoxylin and eosin staining. (b), (d), and (f). Masson's trichrome staining. Black arrow indicates hemorrhage in the lumen of the anus and asterisks indicate hemorrhage in the submucosal tissue. White arrows indicate venous congestion in the spincter muscle layer. Black bar = 1 mm.

Compared with the anorectal tissues of normal dog (Fig. 3a and b), in the section of the anorectal graft from dog 1, hemorrhage in the lumen of the anus and submucosal tissue with arteriovenous extension was observed (Fig. 3c). Dog 1 was scored semi quantitatively as 1.6 ± 0.5 points. Furthermore, severe atrophy of the internal anal sphincter and interstitial expansion were seen in the submucosal tissue compared to the control (Fig. 3d). In the section of the anorectal graft from dog 4, necrosis was not observed, but mild atrophy of the internal anal sphincter and interstitial expansion were found in the submucosal tissue compared to the control (Fig. 3e and f). Dog 4 was scored semi quantitatively as 2.6 ± 0.5 points.

## Discussion

Anorectal allotransplantation was first attempted at St. Mark's Hospital, UK in 2000^18^. They transplanted the anorectal segment from female pigs to male pigs without immunosuppression and achieved successful IMA, IMV, and PNs anastomosis. Histological examination revealed satisfactory inferior mesenteric flow at 24 h after surgery; however, more detailed information regarding this is unavailable. Recently, Galvão et al. reported the development of a rat intestinal transplantation model (including the small and large bowel, rectum, and anus) by anastomoses of the recipient's aorta to the donor's aorta, and the donor's portal vein to the recipient's superior mesenteric vein^16^. Further, they succeeded in swine anorectal autotransplantation by anastomoses between the IMA and the aorta, the IMV and vena cava, and the PNs. No problems regarding graft perfusion were histologically observed two hours postoperatively^19^. We also tried to establish a canine anal autotransplantation model by anastomoses of the IMA, the IMV, and the PNs; however, this failed because of graft necrosis due to blood flow disorder^20^.

In this study, we developed a canine anorectal transplantation model by anastomoses of the bilateral PAs, PVs, and PNs with the non-laparotomy approach. This model is considered important due to three reasons. First, it is important to use dogs as an experimental model because they are as suitable as humans for the observation of defecation control. They can endure defecation desire and defecate at appropriate places as a result of training. In addition, they have vessel sizes and hemodynamic parameters that approximate those of human vessels^23^. Non-human primates are alternative animals for preclinical study, but they are not usually trained with good lavatory manners. Thus, performing such experiments in a canine model is necessary for the study of anal reconstruction. Second, our surgical procedure with the non-laparotomy approach is less invasive and sufficient to observe the anorectal graft perfusion after transplantation. One case of four resulted in venous congestion due to technical error or potentially due to instability of venous flow. Though only dog 1 was female, the anatomical location of PAs, PVs, and PNs were similar to male and didn't impact the surgical approach. More care regarding venous flow may be required because hemorrhoidal problems tend to occur around the anorectal region. Our previous canine data showed the PAs and the PVs had more blood flow toward the anal area compared to the IMA and the IMV^20^. This model without laparotomy is not appropriate for large anorectal defects such as congenital anal dysfunction or abdominoperineal excision, but is well suited for small defects such as intractable anal fistula, trauma, or anal cancer resection. Moreover, in our model, the PAs and the PVs that directly nourish the PNs were anastomosed. These are known to promote nerve and muscle regeneration to maintain blood flow of vessels nourishing the nerve^24^. This has not been discussed extensively in existing literature, but it is very important for long-term anal function convalescence. Third, we initially confirmed graft perfusion for up to 9 days after surgery by ICG fluorescence angiography and histological examination. Although observation was only performed at two and twenty-four hours after surgery using swine models in the past^18^,^19^, it is usually necessary to observe the graft perfusion for a minimum of one week. It is difficult to tell whether or not the autograft that survived is of any functional significance. However we believe our information is very useful for future anorectal transplantation research. Because we confirmed the partial reversal of anorectal function in another canine experiment model (Nishizawa et al., in preparation). Next, the functional recovery of the transplanted anorectal graft and long-term follow-up studies are required after the anorectal allotransplantation. In parallel, experimental human cadaver study needs to go on^25^.

Indications for anorectal transplantation remain controversial because it is not performed for patient survival, but rather to improve QOL, such as is the case for transplantations of the face, larynx, trachea^26^, hand, and uterus. For example, laryngeal transplantation is similar to anorectal transplantation in that it needs postoperative functional recovery with the anastomoses of vessels and nerves. This surgical technique was developed based on anatomy and blood supply using a canine model around 1970^27^ and applied to humans in conjunction with advanced immunosuppressants^15^. Anorectal transplantation may require similar steps to gain public consensus. These kinds of organ/tissue transplantation are accompanied by issues regarding surgical technique, control of rejection and infection, and ethics. Although further animal studies are necessary before clinical application, our established model may aid the advancement of anorectal transplantation medicine.

## Author Contributions

J.A. and Y.N. conceived and designed the experiments. J.A. and Y.N. performed the experiments. T.N., T.S. and K.T. supported the experiments. J.A. and M.N. wrote the main manuscript text and prepared figures 1–3. N.H. and S.H. performed the histological examination. All authors reviewed the manuscript.

## Supplementary Material

Supplementary Information Supplementary Information

Supplementary Information Video 1

Supplementary Information Video 2

Supplementary Information Video 3

## Figures and Tables

**Table 1:** Time Schedule and Surgical Parameters

	Dog 1	Dog 2	Dog 3	Dog 4
Body weight	11 kg	6 kg	8.5 kg	10 kg
Sex	Female	Male	Male	Male
Time for graft harvesting	2 h 00 min	1 h 32 min	2 h 17 min	2 h 55 min
Length of the anorectal graft	5.0 cm	4.5 cm	5.2 cm	5.0 cm
The diameter of the pudendal artery (right/left)	1.5 mm/1.5 mm	2.0 mm/2.0 mm	2.0 mm/1.5 mm	1.5 mm/1.5 mm
The diameter of the pudendal vein (right/left)	2.0 mm/2.0 mm	3.0 mm/2.0 mm	3.5 mm/2.5 mm	1.5 mm/2.0 mm
The diameter of the pudendal nerve (right/left)	2.0 mm/2.0 mm	2.0 mm/3.0 mm	1.5 mm/3.0 mm	1.5 mm/1.5 mm
Time required for neurovascular anastomosis	3 h 07 min	2 h 43 min	3 h 49 min	3 h 53 min
Total ischaemic time	4 h 51 min	3 h 50 min	5 h 08 min	4 h 40 min
Total operation time	8 h 20 min	6 h 59 min	8 h 40 min	8 h 19 min

## Notes

**Grant numbers and sources of support:** This work was supported by grants from Kawano MasanoriMemorial Foundation for Promotion of Pediatrics, the 106^th^ Annual Congress of JSS Memorial Surgical Research Fund and JSPS KAKENHI Grant Number 24-5321.
