# Supplementary material for: Anorectal autotransplantation in a canine model: the first successful report in the short term with the non-laparotomy approach
Source: Sci Rep. 2014 Sep 10;4:6312. doi: 10.1038/srep06312 (PMC4159625; doi:10.1038/srep06312)
Supplement: Supplementary Information [file srep06312-s1.pdf]

# Title: Anorectal autotransplantation in a canine model: the first successful report in the short term with the non-laparotomy approach

Jun Araki,[1,*] Yuji Nishizawa,[2,*] Tatsuo Nakamura,[3] Tomoyuki Sato,[4] Munekazu Naito,[5] Naoyuki Hatayama,[5] Shuichi Hirai,[5] Kensuke Tashiro,[1] Isao Koshima,[1]

## Supplementary information

### Postoperative indocyanine green fluorescence (ICG) angiography of the anorectal graft

Video S1: Normal dog

Video S2: Dog 1 (Unsuccessful case)

Video S3: Dog 4 (Successful case)
